# Supplementary material for: High density oilfield wastewater disposal causes deeper, stronger, and more persistent earthquakes
Source: Nat Commun. 2019 Jul 16;10:3077. doi: 10.1038/s41467-019-11029-8 (PMC6635411; doi:10.1038/s41467-019-11029-8)
Supplement: Supplementary file 1 — Supplementary Information [file 41467_2019_11029_MOESM1_ESM.pdf]

Supplementary Information for

High density oilfield wastewater disposal causes deeper, stronger, and more persistent earthquakes

Pollyea et al.

Supplementary Figures

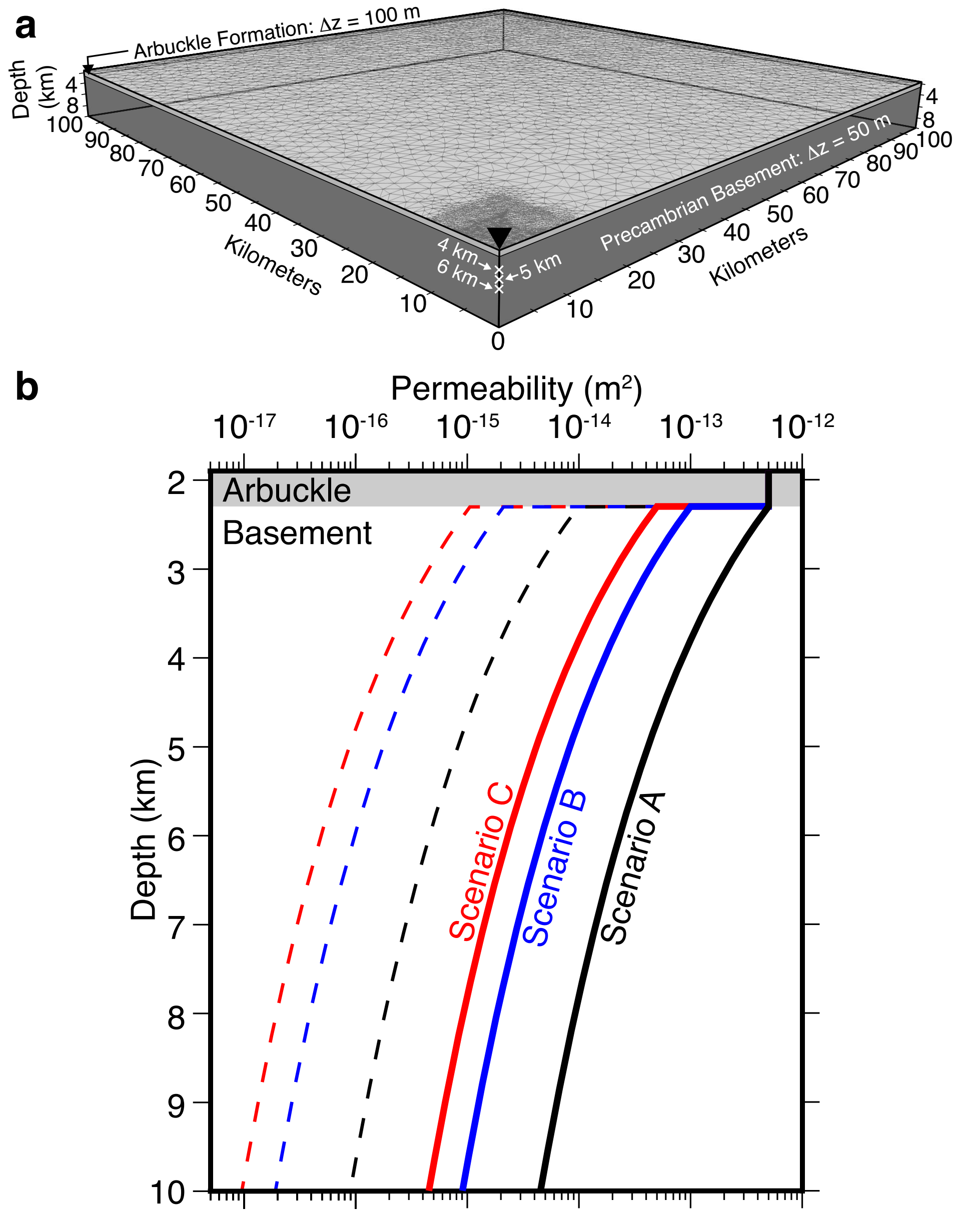

Supplementary Figure 1: Schematic illustration of model domain and permeability structure utilized for this study. a) Lateral grid discretization as the wireframe, and vertical discretization (Δz) is labeled. Triangle denotes well location. The $\times$ symbols denote monitoring locations at 4, 5, and 6 km depth for time-series results. The model domain implements of four-fold symmetry, thus (1) no-flow boundaries are specified in the xz- and yz-planes through the origin and (2) the SWD rate is one-fourth of the total. b) Three permeability scenarios are implemented for this study. Scenario A is the permeability scenario discussed in the main text. Permeability scenarios B and C were simulated to quantify the effects of lower fracture permeability in the Precambrian basement. Solid lines denote fracture permeability and dashed lines denote effective permeability of the combined fracture and matrix continua. Effective permeability is calculated as a volume-weighted arithmetic average.

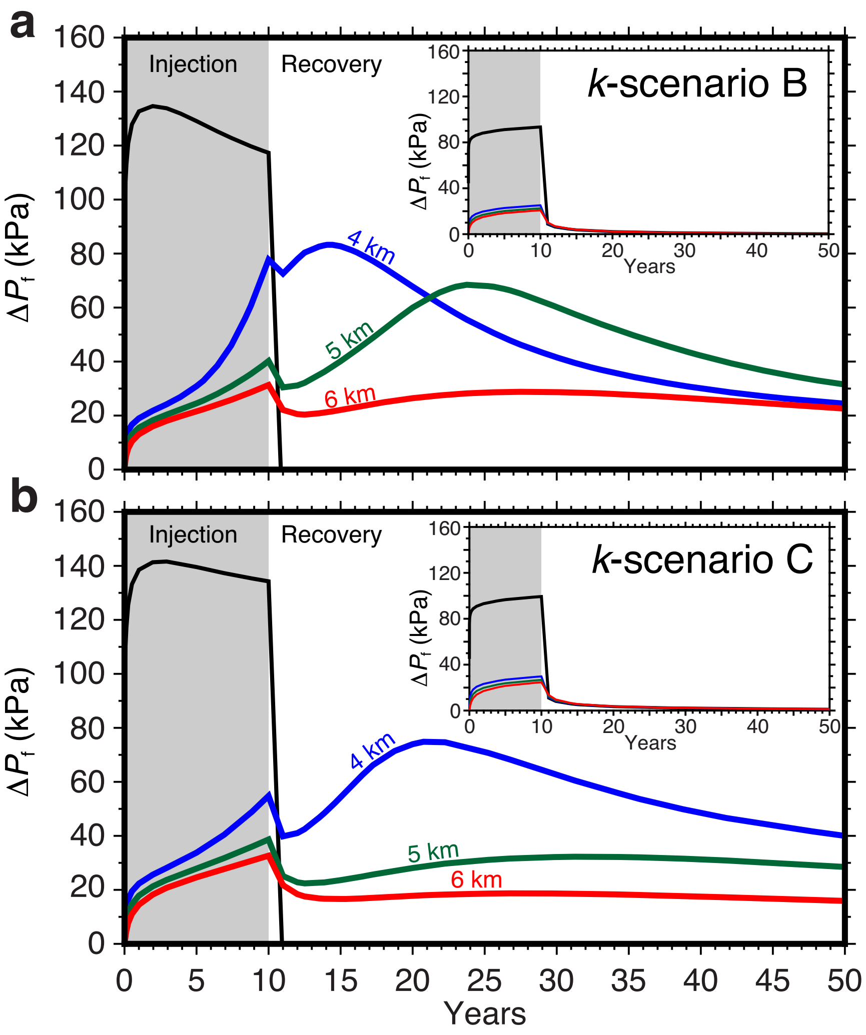

Supplementary Figure 2: Time series of fluid pressure above initial conditions (ΔP_f) for variable density models using (a) permeability scenario B and (b) permeability scenario C. ΔP_f is tracked at monitoring points located within the SWD well (black) and directly below the well at 4 km (blue), 5 km (green), and 6 km (red) depth. Insets are ΔP_f for corresponding constant density models. For a given depth, the difference between ΔP_f in main panel and inset are due to the advective transport of high density wastewater.

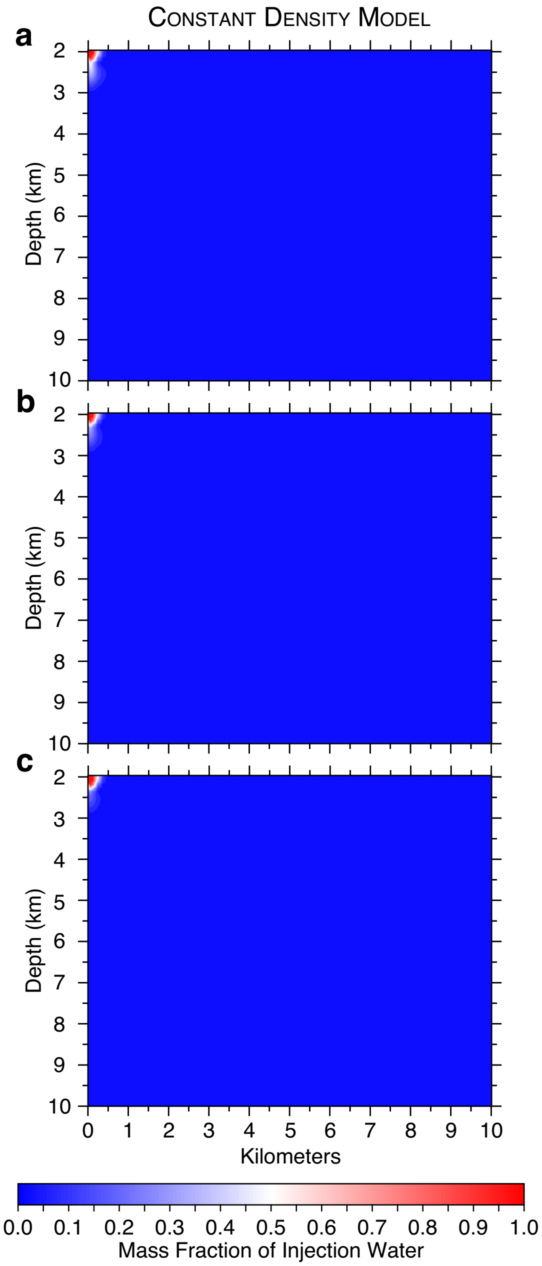

Supplementary Figure 3: Simulation results for the recovery phase of the constant density model using permeability Scenario A following 10 years of salt water dispsoal (SWD) at 2,080 m^3 day^{-1}. There are no ΔP_f contours because results for (a) 1 year, (b) 5 years, and (c) 10 years of post-injection recovery show that fluid pressure rapidly returns to background conditions when there is no density differential between wastewater and host rock fluids. Shading is mass fraction of injected water.

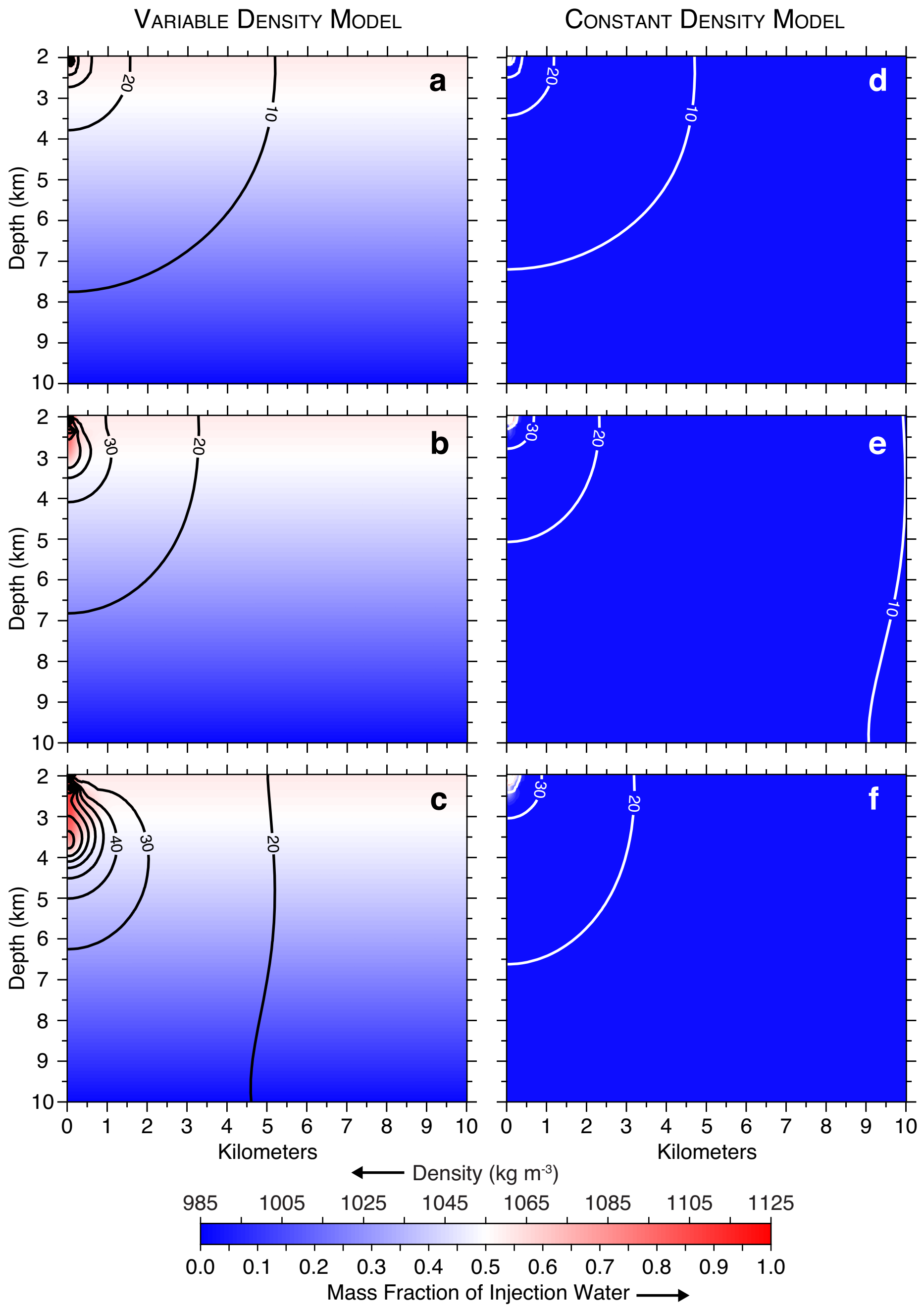

Supplementary Figure 4: Simulation results for permeability scenario B after 10 years of SWD at 2,080 m^3 day^{-1}. Left column is variable density model after (a) 1 year, (b) 5 years, and (c) 10 years of SWD. Black contour lines are fluid pressure change above initial conditions in 10 kPa intervals and shading is fluid density. Right column is constant density model after (d) 1 year, (e) 5 years, and (f) 10 years of SWD. White contour lines are fluid pressure change above initial conditions in 10 kPa intervals and shading is mass fraction of injected water.

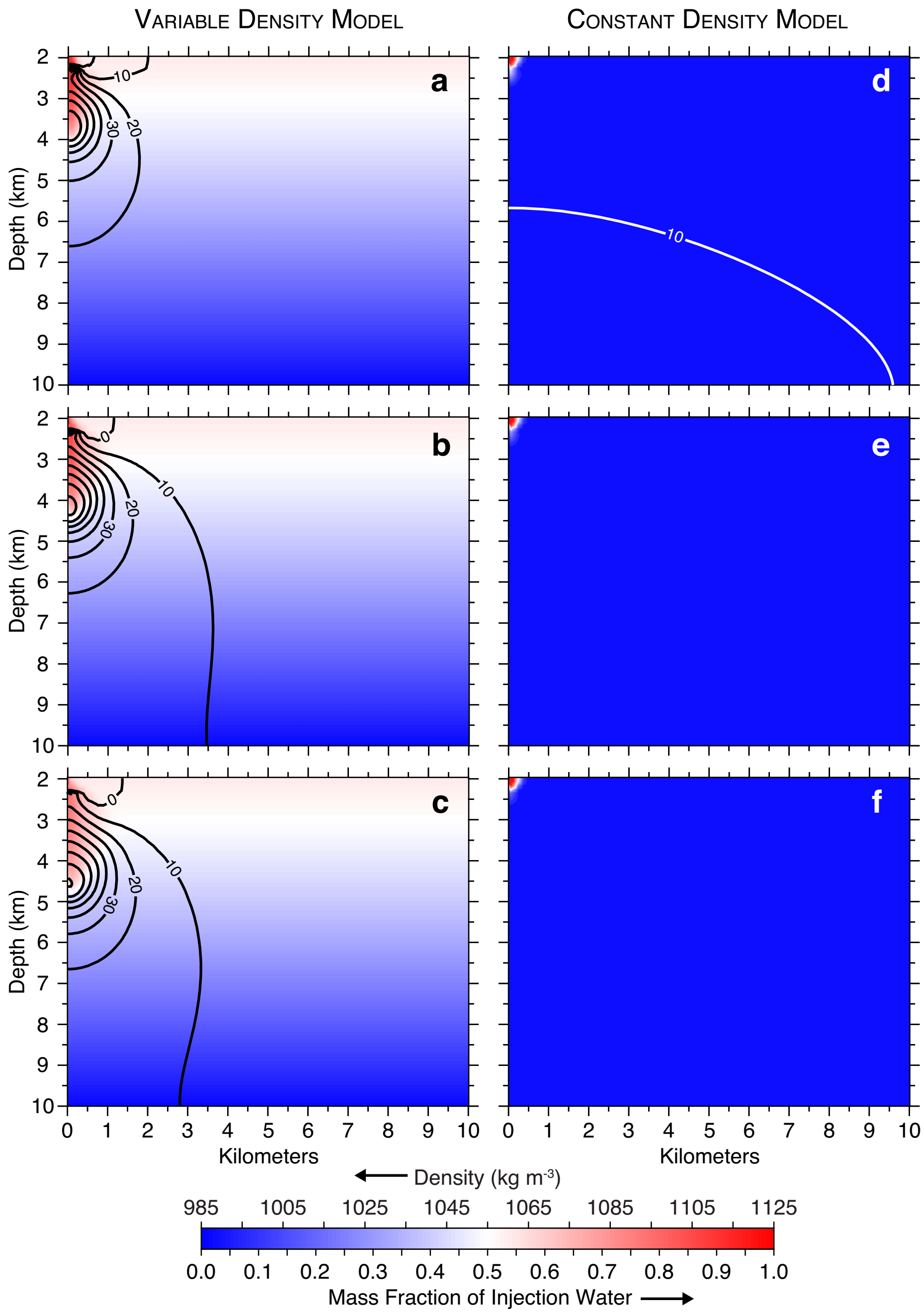

Supplementary Figure 5: Post-injection pressure recovery for permeability scenario B. Left column is variable density model for (a) 1 year, (b) 5 years, and (c) 10 years of post-injection recovery. Black contour lines are fluid pressure change above pre-injection conditions in 10 kPa intervals and shading is fluid density. Right column is constant density model after (d) 1 year, (e) 5 years, and (f) 10 years of post-injection recovery. White contour lines are fluid pressure change above pre-injection conditions in 10 kPa intervals and shading is mass fraction of injected water.

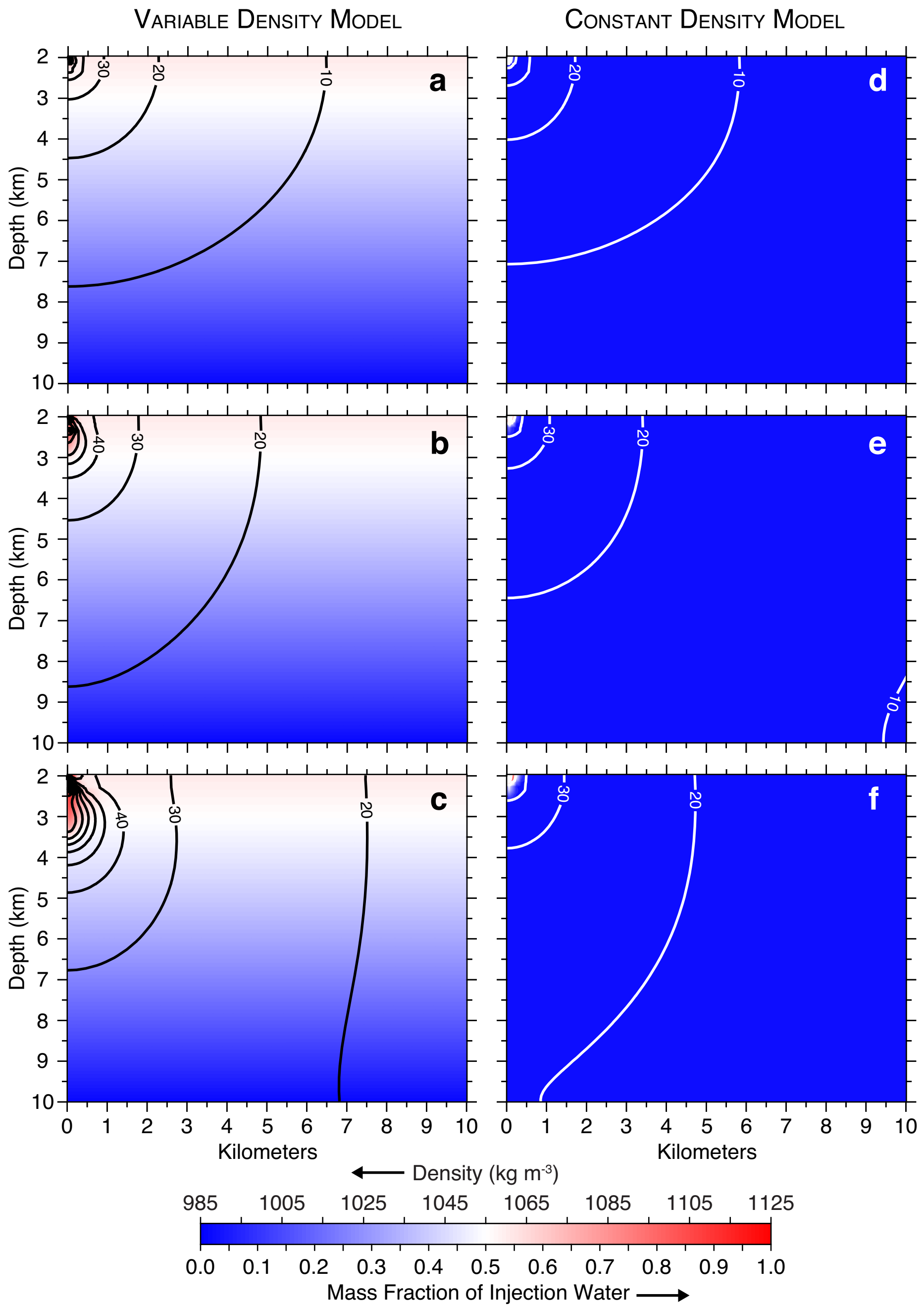

Supplementary Figure 6: Simulation results for permeability scenario C after 10 years of SWD at 2,080 m^3 day^{-1}. Left column is variable density model after (a) 1 year, (b) 5 years, and (c) 10 years of SWD. Black contour lines are fluid pressure change above initial conditions in 10 kPa intervals and shading is fluid density. Right column is constant density model after (d) 1 year, (e) 5 years, and (b) 10 years of SWD. White contour lines are fluid pressure change above initial conditions in 10 kPa intervals and shading is mass fraction of injected water.

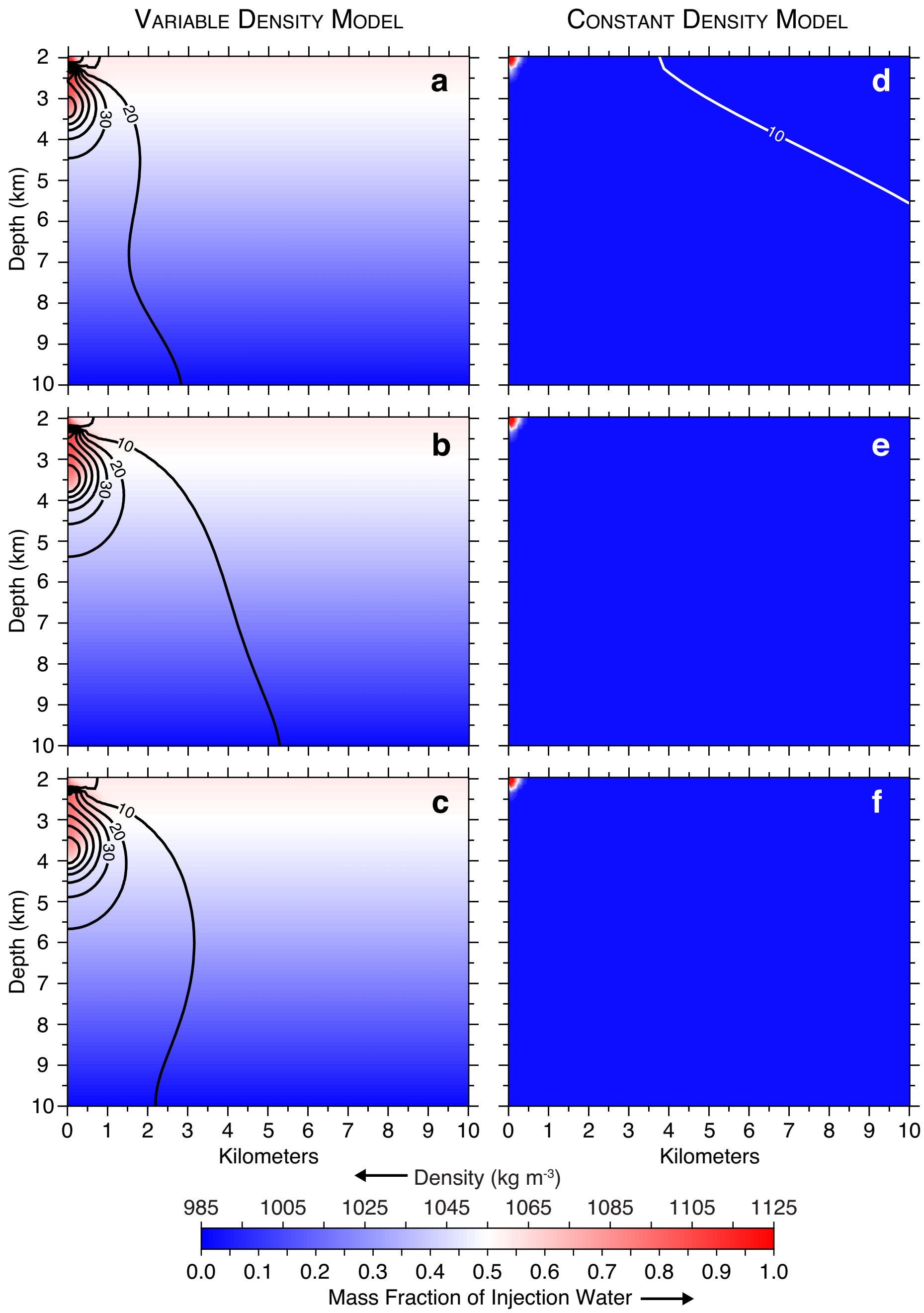

Supplementary Figure 7: Post-injection pressure recovery for permeability scenario C. Left column is variable density model for (a) 1 year, (b) 5 years, and (c) 10 years of post-injection recovery. Black contour lines are fluid pressure change above pre-injection conditions in 10 kPa intervals and shading is fluid density. Right column is constant density model after (d) 1 year, (e) 5 years, and (f) 10 years of post-injection recovery. White contour lines are fluid pressure change above pre-injection conditions in 10 kPa intervals and shading is mass fraction of injected water.

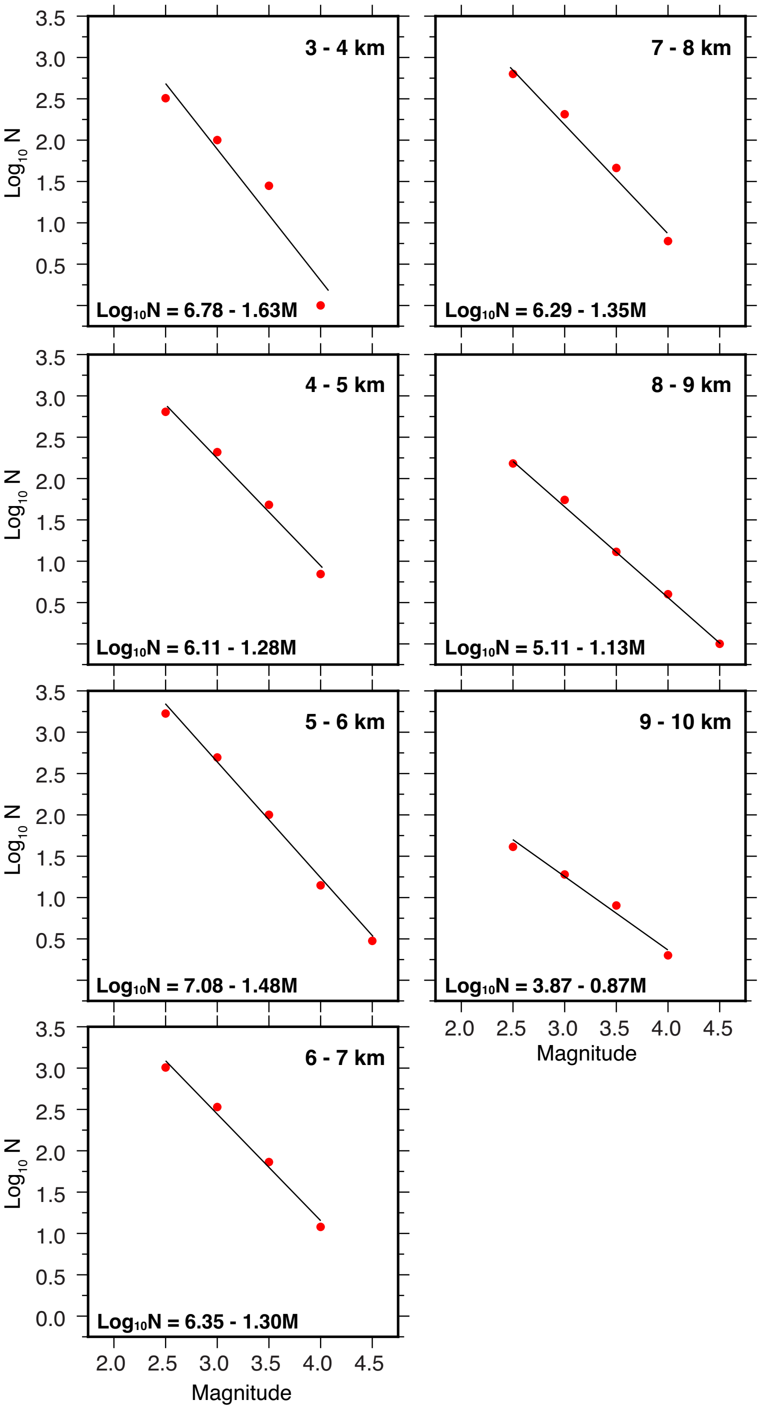

Supplementary Figure 8: Earthquake frequency-magnitude plots at 1 km depth intervals between 3 and 10 km depth within north-central Oklahoma and southern Kansas for time period 1 January 2013 through 31 December 2018. Solid black lines are fit by ordinary least squares regression. For each depth interval, the slope of the regression line is the *b*-value.

Supplementary Tables

Supplementary Table 1: Hydraulic, thermal, and geochemical properties utilized for the model scenario.

Parameter	Value	Units
Permeability[1]		
Arbuckle	5×10^{-13}	m^2
Basement Matrix	1×10^{-20}	m^2
Basement Fracture	Sup. Fig. 1	m^2
Porosity		
Arbuckle	0.10	–
Basement Matrix	0.02	–
Basement Fracture	0.10	–
Rock Density		
Arbuckle	2,500	kg m^{-3}
Basement	2,800	kg m^{-3}
Compressibility		
Arbuckle	1.7×10^{-10}	Pa^{-1}
Basement Matrix	4.5×10^{-11}	Pa^{-1}
Basement Fracture	4.5×10^{-11}	Pa^{-1}
Thermal Properties[2]		
Conductivity	2.2	W m^{-1} $°C^{-1}$
Heat Capacity	1,000	J kg^{-1} $°C^{-1}$
Diffusion Coefficients		
Brine	1.14×10^{-9}	m^2 s^{-1}
Pure Water	2.30×10^{-9}	m^2 s^{-1}

[1] All permeability is isotropic, $k_x = k_y = k_z$.

[2] Arbuckle and basement.
